# Supplementary material for: A pilot study of bladder voiding with real-time MRI and computational fluid dynamics
Source: PLoS One. 2020 Nov 19;15(11):e0238404. doi: 10.1371/journal.pone.0238404 (PMC7676741; doi:10.1371/journal.pone.0238404)
Supplement: Attachment — Submitted filename: Review PLOSone Article.docx [file pone.0238404.s003.docx]

**Review PLOSone Article**

**Methods:**

1. Authors should include how they documented patients in control group were asymptomatic (e.g. International Prostate Symptom Score or AUA symptom score).

2. How were the 3 BPH/LUTS patients selected from the urology clinic? (Randomly?, IPSS?, Traditional Urodynamics?)

3. Patient selection did not specifically exclude those with neurologic diseases that are known to effect bladder function, such as, diabetes, parkinsonism, etc., even though the control group showed no symptoms. The group with BPH/LUTS could also have complicating neurologic disorders. Therefore, I suggest that the authors specifically state that there were no histories of such diseases.

4. It would have been nice to have Total Prostatic Volumes and Transition zone volumes on at least the BPH/LUTS group.

5. Examination with patients in the supine position in not very physiologic and represents a distinct limitation of the experiment. (Unlike with cardiac CFD.) The potential effect of positioning should be brought up in the DISCUSSION section.

6. The models rely on many assumptions, some of which the authors accurately report

**Results:**

1. P9,line1 Statement should insert the words “may be” before “due” or amend to say “there is an association between wall displacement and increased flow velocity in the bladder”, since they have not done a large cohort to provide proof.

2. Fig. 6 Caption The authors might have provided more information on how to interpret “streaming lines” and “time points.” Engineers may be familiar with these concepts, but urologists, radiologists imagers and other readers probably need assistance. Also, “velocity magnitude” should be defined here or in the text of the manuscript.

3. Fig. 7 Caption Insert “likely” before “due”.

**Discussion:**

1. P10,line 3 The authors are “profoundly startled” by dome movement, but I would expect that because the base of the inferior detrusor is limited in movement by the thicker and stiffer stiffer overlying trigone. This would be expected to be exaggerated by gravity if the subject voids in the usual upright posture. A few examples of this are listed below:

## [[Assessment of movements of the different anatomic portions of the bladder, implications for image-guided radiation therapy for bladder cancer]. [French]](http://ovidsp.dc2.ovid.com.ezp1.lib.umn.edu/ovid-a/ovidweb.cgi?&S=FOACFPDHOBEBIIEBIPAKIFOGFMPNAA00&Complete+Reference=S.sh.55%7c21%7c1&Counter5=SS_view_found_complete%7c22365260%7cppezv%7cmedline%7cmed9&Counter5Data=22365260%7cppezv%7cmedline%7cmed9)

Evaluation des mouvements des differentes portions anatomiques de la vessie, implications pour la radiotherapie guidee par l'image pour les cancers de vessie.

Pan Q; Thariat J; Bogalhas F; Lagrange JL.

*Cancer Radiotherapie. 16(3):167-78, 2012 May.*

*[Journal Article]*

**UI:** 22365260

**Authors Full Name**
Pan, Q; Thariat, J; Bogalhas, F; Lagrange, J-L.

[<http://ovidsp.dc2.ovid.com.ezp1.lib.umn.edu/ovid-a/ovidweb.cgi?&S=FOACFPDHOBEBIIEBIPAKIFOGFMPNAA00&Get+Citation=S.sh.55|21>Cite](http://ovidsp.dc2.ovid.com.ezp1.lib.umn.edu/ovid-a/ovidweb.cgi?&S=FOACFPDHOBEBIIEBIPAKIFOGFMPNAA00&Get+Citation=S.sh.55%7c21)[<http://ovidsp.dc2.ovid.com.ezp1.lib.umn.edu/ovid-a/ovidweb.cgi?&S=FOACFPDHOBEBIIEBIPAKIFOGFMPNAA00&FormProcessor=item__add&InputArgs=form_name:item__add|interface_origin:oi|keep_selection:Y|display_type:Titles|dlname:S.sh.55|start:21|count:10|index:21|on_mm_page:|&Counter5=SS_add_to_my_projects|22365260|ppezv|medline|med9> My Projects](http://ovidsp.dc2.ovid.com.ezp1.lib.umn.edu/ovid-a/ovidweb.cgi?&S=FOACFPDHOBEBIIEBIPAKIFOGFMPNAA00&FormProcessor=item__add&InputArgs=form_name%3aitem__add%7cinterface_origin%3aoi%7ckeep_selection%3aY%7cdisplay_type%3aTitles%7cdlname%3aS.sh.55%7cstart%3a21%7ccount%3a10%7cindex%3a21%7con_mm_page%3a%7c&Counter5=SS_add_to_my_projects%7c22365260%7cppezv%7cmedline%7cmed9)[<http://ovidsp.dc2.ovid.com.ezp1.lib.umn.edu/ovid-a/ovidweb.cgi> Annotate](http://ovidsp.dc2.ovid.com.ezp1.lib.umn.edu/ovid-a/ovidweb.cgi)

AB PURPOSE: To assess interfraction and intrafraction bladder **wall** movements in the different anatomic portions of the bladder. PATIENTS AND METHODS: Six patients were treated for prostate cancer with conformal irradiation. Daily online cone beam computed tomography was performed for repositioning and an additional one was performed following irradiation once weekly. Four craniocaudal levels were defined to calculate movements amplitudes compared to the scanner tracking: level 1 at the bladder neck, level 2 at mid-height of the bladder, level 3 at mid-height of the dome, level 4 at the apex in a distended bladder. Bladder height was also measured. RESULTS: On 198 daily cone beam computed tomographies, radial bladder right/left/anterior/posterior **wall** displacements at level 2 were 0.08 +/- 0.24, 0.11 +/- 0.33, 0.16 +/- 0.45 and 0.14 +/- 0.50 cm and at level 3 0.07 +/- 0.78, 0.18 +/- 0.98, 0.43 +/- 0.94 and 0.04 +/- 1.02 cm. Dome and neck displacements were 0.08 +/- 1.41 cm and 0.08 +/- 0.64 cm. Seventeen cone beam computed tomographies were done following irradiation. Radial bladder right/left/anterior/halfway up the trine **wall** displacements at level 2 before and after irradiation were 0.02+/-0.18, 0.01+/-0.30, 0.09 +/- 0.32 and 0.22 +/- 0.42 cm and at level 3 0.27 +/- 0.60, 0.37 +/- 1.15, 0.18 +/- 0.87 and 0.54 +/- 1.68 cm. CONCLUSION: Significant bladder **wall** displacements were observed on the anterior **wall** and upper portion of the bladder. Isotropic margins may not be sufficient to account for inter- and intrafraction bladder **wall** displacements at the latter levels. Tailored bladder anatomy-based anisotropic margins may be necessary to optimally spare the small intestine and to guaranty proper tumour coverage in case of bladder cancer. For upper bladder tumours, margins of over 2 cm would be necessary, which make them less adequate for external beam irradiation.

### [Assessment of Bladder Motion for Clinical Radiotherapy Practice Using Cine–Magnetic Resonance Imaging](https://primo.lib.umn.edu/primo-explore/fulldisplay?docid=RS_6036030166648211magneticresonanceimaging&context=SP&vid=TWINCITIES&lang=en_US)

Catherine A McBain;Vincent S Khoo;David L Buckley;Jonathan S Sykes;Melanie M Green;Richard A Cowan;Charles E Hutchinson;Christopher J Moore;Patricia M Price

**ISSN:** 0360-3016; **DOI:** 10.1016/j.ijrobp.2008.11.040

International journal of radiation oncology, biology, physics. , 2009, Vol.75(3),

2. p10 line 7 “thatt” typo

3. p10,l para 7 The authors might consider that CFD may also show vesicoureteral reflux, although it is not demonstrated in this study cohort.

**REFERENCES: There are several incomplete or mistaken references**

#13 Busse R incomplete reference

#28 Turkiye “Klinikleri” J Med Sci

**SUMMARY**

Pilot feasibility studies require a lesser level of evidence than more determinative research. The authors conceive of an image-based non-invasive technique that may substitute for traditional multichannel urodynamics. I would offer that their studies may provide different information rather than fully replace pressure-flow studies. The results of this study can make no conclusions other than proof of concept which it adequately does. I would recommend the authors re-phrase the final paragraph to say that the findings “in men with BPH/LUTS “suggest asymmetric bladder wall motion compared to healthy men…….”
