# Supplementary material for: A pilot study of bladder voiding with real-time MRI and computational fluid dynamics
Source: PLoS One. 2020 Nov 19;15(11):e0238404. doi: 10.1371/journal.pone.0238404 (PMC7676741; doi:10.1371/journal.pone.0238404)
Supplement: Attachment — Submitted filename: Sub3_CFD_Bladder_response.docx [file pone.0238404.s004.docx]

**We thank the reviewers for their consideration of our manuscript and for their expert advice on how to best improve our manuscript. Changes to the manuscript are tracked and point-by-point responses to reviewer comments are below.**

**Methods:**

1. Authors should include how they documented patients in control group were asymptomatic (e.g. International Prostate Symptom Score or AUA symptom score).

**Healthy controls were recruited from a database in the department of Radiology. At time of consenting patients were asked about LUT symptoms.**

**“At the time of consenting it was confirmed that patients were not experiencing LUT symptoms.”**

2. How were the 3 BPH/LUTS patients selected from the urology clinic? (Randomly?, IPSS?, Traditional Urodynamics?)

**Patients with known BPH/LUTS and scheduled for surgical treatment were recruited for the study.**

3. Patient selection did not specifically exclude those with neurologic diseases that are known to effect bladder function, such as, diabetes, parkinsonism, etc., even though the control group showed no symptoms. The group with BPH/LUTS could also have complicating neurologic disorders. Therefore, I suggest that the authors specifically state that there were no histories of such diseases.

**None of the patients had a history of neurological disease**

**“Three men with known BPH/LUTS who were scheduled for surgical treatment were recruited from the University of Wisconsin Urology clinic (ages 73, 71, and 54), none of whom had a history of neurological disease. The inclusion criteria were adult men recently diagnosed with BPH.”**

4. It would have been nice to have Total Prostatic Volumes and Transition zone volumes on at least the BPH/LUTS group.

**Prostate volumes are now included in the results section.**

**“Prostate volumes segmented from 3D FSE MRI were larger for BPH/LUTS patients (45, 80, and 106 mL) compared to healthy volunteers (18, 37, and 42 mL).”**

5. Examination with patients in the supine position in not very physiologic and represents a distinct limitation of the experiment. (Unlike with cardiac CFD.) The potential effect of positioning should be brought up in the DISCUSSION section.

**The potential effects of positioning are now discussed In the manuscript.**

**“Men normally void either standing or sitting down, and it would be ideal to perform the dynamic studies in a standing position, however, that is not possible due to the patient’s position in the MRI scanner. While multichannel urodynamic evaluation is usually performed with men either standing or sitting, it is often performed in supine position for patients with neurologic disease such as spinal cord injury. Previous studies have demonstrated modest effects of study position on quantitative measures of voiding pressure and urine flow, and ; however, there is no evidence that the anatomy and contractile function of the bladder are significantly altered by position.”**

6. The models rely on many assumptions, some of which the authors accurately report

**Additional assumptions are now reported.**

**“The urethra wall was assumed to be rigid and the urethra outlet was set to atmospheric pressure. Additionally, the urine density and viscosity were assumed to be the same for all subjects.”**

**Results:**

1. P9,line1 Statement should insert the words “may be” before “due” or amend to say “there is an association between wall displacement and increased flow velocity in the bladder”, since they have not done a large cohort to provide proof.

**“Urodynamics results from computational fluid dynamics (CFD) are shown in Figure 6 for a sagittal plane near the center of the bladder. Control subjects had higher urine velocities in the bladder than men with BPH/LUTS that may be due to their greater bladder wall displacements.”**

2. Fig. 6 Caption The authors might have provided more information on how to interpret “streaming lines” and “time points.” Engineers may be familiar with these concepts, but urologists, radiologists imagers and other readers probably need assistance. Also, “velocity magnitude” should be defined here or in the text of the manuscript.

**“CFD results showing velocity contours and streamlines on a sagittal plane at the center of the bladder for each subject. Results are displayed for time frames from near both the initiation and termination voiding. Streamlines indicate the direction of urine flow and velocity magnitude is the magnitude of the velocity vector or the overall speed of urine flow.”**

3. Fig. 7 Caption Insert “likely” before “due”.

**“Bladder vorticity was lower for the men with BPH/LUTS, likely due to their lower flowrates and urine velocities. After making vorticity dimensionless (a normalization to account for different flow rates between subjects), vorticity was similar for both groups.”**

**Discussion:**

1. P10,line 3 The authors are “profoundly startled” by dome movement, but I would expect that because the base of the inferior detrusor is limited in movement by the thicker and stiffer stiffer overlying trigone. This would be expected to be exaggerated by gravity if the subject voids in the usual upright posture. A few examples of this are listed below:

## [[Assessment of movements of the different anatomic portions of the bladder, implications for image-guided radiation therapy for bladder cancer]. [French]](http://ovidsp.dc2.ovid.com.ezp1.lib.umn.edu/ovid-a/ovidweb.cgi?&S=FOACFPDHOBEBIIEBIPAKIFOGFMPNAA00&Complete+Reference=S.sh.55%7c21%7c1&Counter5=SS_view_found_complete%7c22365260%7cppezv%7cmedline%7cmed9&Counter5Data=22365260%7cppezv%7cmedline%7cmed9)

Evaluation des mouvements des differentes portions anatomiques de la vessie, implications pour la radiotherapie guidee par l'image pour les cancers de vessie.

Pan Q; Thariat J; Bogalhas F; Lagrange JL.

*Cancer Radiotherapie. 16(3):167-78, 2012 May.*

*[Journal Article]*

**UI:** 22365260

**Authors Full Name**
Pan, Q; Thariat, J; Bogalhas, F; Lagrange, J-L.

[<http://ovidsp.dc2.ovid.com.ezp1.lib.umn.edu/ovid-a/ovidweb.cgi?&S=FOACFPDHOBEBIIEBIPAKIFOGFMPNAA00&Get+Citation=S.sh.55|21>Cite](http://ovidsp.dc2.ovid.com.ezp1.lib.umn.edu/ovid-a/ovidweb.cgi?&S=FOACFPDHOBEBIIEBIPAKIFOGFMPNAA00&Get+Citation=S.sh.55%7c21)[<http://ovidsp.dc2.ovid.com.ezp1.lib.umn.edu/ovid-a/ovidweb.cgi?&S=FOACFPDHOBEBIIEBIPAKIFOGFMPNAA00&FormProcessor=item__add&InputArgs=form_name:item__add|interface_origin:oi|keep_selection:Y|display_type:Titles|dlname:S.sh.55|start:21|count:10|index:21|on_mm_page:|&Counter5=SS_add_to_my_projects|22365260|ppezv|medline|med9> My Projects](http://ovidsp.dc2.ovid.com.ezp1.lib.umn.edu/ovid-a/ovidweb.cgi?&S=FOACFPDHOBEBIIEBIPAKIFOGFMPNAA00&FormProcessor=item__add&InputArgs=form_name%3aitem__add%7cinterface_origin%3aoi%7ckeep_selection%3aY%7cdisplay_type%3aTitles%7cdlname%3aS.sh.55%7cstart%3a21%7ccount%3a10%7cindex%3a21%7con_mm_page%3a%7c&Counter5=SS_add_to_my_projects%7c22365260%7cppezv%7cmedline%7cmed9)[<http://ovidsp.dc2.ovid.com.ezp1.lib.umn.edu/ovid-a/ovidweb.cgi> Annotate](http://ovidsp.dc2.ovid.com.ezp1.lib.umn.edu/ovid-a/ovidweb.cgi)

AB PURPOSE: To assess interfraction and intrafraction bladder **wall** movements in the different anatomic portions of the bladder. PATIENTS AND METHODS: Six patients were treated for prostate cancer with conformal irradiation. Daily online cone beam computed tomography was performed for repositioning and an additional one was performed following irradiation once weekly. Four craniocaudal levels were defined to calculate movements amplitudes compared to the scanner tracking: level 1 at the bladder neck, level 2 at mid-height of the bladder, level 3 at mid-height of the dome, level 4 at the apex in a distended bladder. Bladder height was also measured. RESULTS: On 198 daily cone beam computed tomographies, radial bladder right/left/anterior/posterior **wall** displacements at level 2 were 0.08 +/- 0.24, 0.11 +/- 0.33, 0.16 +/- 0.45 and 0.14 +/- 0.50 cm and at level 3 0.07 +/- 0.78, 0.18 +/- 0.98, 0.43 +/- 0.94 and 0.04 +/- 1.02 cm. Dome and neck displacements were 0.08 +/- 1.41 cm and 0.08 +/- 0.64 cm. Seventeen cone beam computed tomographies were done following irradiation. Radial bladder right/left/anterior/halfway up the trine **wall** displacements at level 2 before and after irradiation were 0.02+/-0.18, 0.01+/-0.30, 0.09 +/- 0.32 and 0.22 +/- 0.42 cm and at level 3 0.27 +/- 0.60, 0.37 +/- 1.15, 0.18 +/- 0.87 and 0.54 +/- 1.68 cm. CONCLUSION: Significant bladder **wall** displacements were observed on the anterior **wall** and upper portion of the bladder. Isotropic margins may not be sufficient to account for inter- and intrafraction bladder **wall** displacements at the latter levels. Tailored bladder anatomy-based anisotropic margins may be necessary to optimally spare the small intestine and to guaranty proper tumour coverage in case of bladder cancer. For upper bladder tumours, margins of over 2 cm would be necessary, which make them less adequate for external beam irradiation.

### [Assessment of Bladder Motion for Clinical Radiotherapy Practice Using Cine–Magnetic Resonance Imaging](https://primo.lib.umn.edu/primo-explore/fulldisplay?docid=RS_6036030166648211magneticresonanceimaging&context=SP&vid=TWINCITIES&lang=en_US)

Catherine A McBain;Vincent S Khoo;David L Buckley;Jonathan S Sykes;Melanie M Green;Richard A Cowan;Charles E Hutchinson;Christopher J Moore;Patricia M Price

**ISSN:** 0360-3016; **DOI:** 10.1016/j.ijrobp.2008.11.040

International journal of radiation oncology, biology, physics. , 2009, Vol.75(3),

**This statement has been removed and we these studies are now included in our discussion section.**

**“Results from daily CTs of bladder cancer patients over the course of a week (presumably at varied levels of bladder filling) similarly show that the bladder wall displacements were greater for the dome of the bladder than the base [20]”**

**“Asymmetric bladder wall motion has previously been identified in bladder cancer patients during bladder filling [26].”**

2. p10 line 7 “thatt” typo

**This typo has been corrected.**

3. p10,l para 7 The authors might consider that CFD may also show vesicoureteral reflux, although it is not demonstrated in this study cohort.

**Investigation of vesicoureteral reflux would require our models to include the ureters. This is now discussed in the manuscript.**

**“Further improvements to the simulation realism by including the ureters could expand CFD analysis to include phenomena such as vesicoureteral reflux.”**

**REFERENCES: There are several incomplete or mistaken references**

#13 Busse R incomplete reference

#28 Turkiye “Klinikleri” J Med Sci

**Reference #13 has been completed. Reference #28 (now #30) has been double checked and it is correct.**

**SUMMARY**

Pilot feasibility studies require a lesser level of evidence than more determinative research. The authors conceive of an image-based non-invasive technique that may substitute for traditional multichannel urodynamics. I would offer that their studies may provide different information rather than fully replace pressure-flow studies. The results of this study can make no conclusions other than proof of concept which it adequately does. I would recommend the authors re-phrase the final paragraph to say that the findings “in men with BPH/LUTS “suggest asymmetric bladder wall motion compared to healthy men…….”

**The summary has been rewritten**

**“In summary, this pilot study demonstrated the feasibility of MRI bladder voiding studies to non-invasively investigate bladder function. Results from displacement analysis suggested that men with BPH/LUTS have decreased and asymmetric bladder wall motion compared to healthy male controls and fluid dynamic analysis of voiding suggested that men with BPH/LUTS have larger recirculation regions in the bladder.”**
