# Supplementary material for: A pilot study of bladder voiding with real-time MRI and computational fluid dynamics
Source: PLoS One. 2020 Nov 19;15(11):e0238404. doi: 10.1371/journal.pone.0238404 (PMC7676741; doi:10.1371/journal.pone.0238404)
Supplement: Attachment — Submitted filename: Reviewer comments.docx [file pone.0238404.s002.docx]

**We thank the reviewer and editor for their consideration and feedback regarding our manuscript. Based on reviewer and editor comments we have expanded our manuscript from a case report of one healthy subject to a small cohort of three healthy subjects and three subjects with lower urinary tract symptoms. As the manuscript has been entirely rewritten changes to the manuscript are not marked. Point by point responses to reviewer comments follow.**

1. Is the manuscript technically sound, and do the data support the conclusions?

   The manuscript must describe a technically sound piece of scientific research with data that supports the conclusions. Experiments must have been conducted rigorously, with appropriate controls, replication, and sample sizes. The conclusions must be drawn appropriately based on the data presented.

   Reviewer #1: Yes

**Agree.**

2. Has the statistical analysis been performed appropriately and rigorously?

Reviewer #1: No

**Given the small sample sizes (n=3 and n=3 for both groups) we did not perform statistical analysis. The purpose of this study was to demonstrate a novel technique to assess bladder function so we feel this is appropriate. Additionally, we have added “pilot study” to the manuscript title per the editors advice and given our small sample size.**

3. Have the authors made all data underlying the findings in their manuscript fully available?

The [PLOS Data policy](http://www.plosone.org/static/policies.action#sharing) requires authors to make all data underlying the findings described in their manuscript fully available without restriction, with rare exception (please refer to the Data Availability Statement in the manuscript PDF file). The data should be provided as part of the manuscript or its supporting information, or deposited to a public repository. For example, in addition to summary statistics, the data points behind means, medians and variance measures should be available. If there are restrictions on publicly sharing data—e.g. participant privacy or use of data from a third party—those must be specified.

Reviewer #1: No

**The data points used to create the figures are now included in a supplementary excel file.**

4. Is the manuscript presented in an intelligible fashion and written in standard English?

PLOS ONE does not copyedit accepted manuscripts, so the language in submitted articles must be clear, correct, and unambiguous. Any typographical or grammatical errors should be corrected at revision, so please note any specific errors here.

Reviewer #1: Yes

**Agree.**

5. Review Comments to the Author

Please use the space provided to explain your answers to the questions above. You may also include additional comments for the author, including concerns about dual publication, research ethics, or publication ethics. (Please upload your review as an attachment if it exceeds 20,000 characters)

Reviewer #1: This is an interesting paper that has applied cardiac wall movement analysis using MRI to assess bladder voiding. The data presented is a bit premature, as the technique was only assessed on one healthy volunteer. There is no statistical analyses to support their hypothesis that most of the bladder wall movement occurs in the dome region. Although the application of the MRI methodology is new for bladder voiding, the approach to measure cardiac wall movement is well documented. I applaud the investigators for attempting to use the methodology in the bladder, which requires such approaches to non-invasively assess its function. Although, the approach is not entirely non-invasive, as the placement of the catheter and voiding approach described seems like it would be cumbersome for patients with bladder disorders.
At the very least, this method needs to be assessed in multiple individuals to support the outcome that there are regional differences in bladder wall movement during voiding. Individuals with bladder voiding issues should have also been included, which would have been more relevant to the urology community.

**We have expanded our manuscript from a case report of one healthy subject to a small cohort of three healthy subjects and three subjects with lower urinary tract symptoms. Given the small sample size we did not perform statistical analysis.**

6. PLOS authors have the option to publish the peer review history of their article ([what does this mean?](https://journals.plos.org/plosone/s/editorial-and-peer-review-process#loc-peer-review-history)). If published, this will include your full peer review and any attached files.


If you choose “no”, your identity will remain anonymous but your review may still be made public.


**Do you want your identity to be public for this peer review?** For information about this choice, including consent withdrawal, please see our [Privacy Policy](https://www.plos.org/privacy-policy).

Reviewer #1: No

**We do not wish to make the peer review history public.**
